# Numerical Study of Tangential Traction Mechanism between Pattern Blocks of Agricultural Radial Tires and Soft Soil

**DOI:** 10.3390/ma17163906

**Published:** 2024-08-07

**Authors:** Sheng Li, Jian Wu, Yang Wan, Benlong Su, Youshan Wang

**Affiliations:** 1 Center for Rubber Composite Materials and Structures, Harbin Institute of Technology, Weihai 264209, China; lisheng072230@gmail.com (S.L.); wanyang24@163.com (Y.W.); subenlong@hit.edu.cn (B.S.); wangys@hit.edu.cn (Y.W.); 2 National Key Laboratory of Science and Technology on Advanced Composites in Special Environments, Harbin Institute of Technology, Harbin 150090, China

**Keywords:** traction performance, contact behavior, tire–soil interface, pattern blocks, FEM, soil shear force

## Abstract

With the increasing requirements of agricultural machinery, the study of the contact relationship between the tire–soil interface and the improvement of traction efficiency has gradually become a main concern. In this study, the pattern on the agricultural tire was simplified into single-pitch pattern blocks. The pattern blocks were made of rubber material that was highly resistant to abrasion and bending. The experiment was carried out by pressing the three types of patterned block construction into the soil and the pure sliding under the soil. The simulation used the Coupled Eulerian–Lagrangian Method (CEL) to verify the experimental results. We found that the herringbone pattern block was subjected to the highest stress for the same depth of downward pressure. The horizontal force generated by the pure sliding was also the highest. The results showed that the numerically simulated and experimentally measured data exhibited similar trends and average values. In addition, the increase in the contact area between the tire and the soil reduced the compaction and sinking of the soil. The herringbone pattern structure not only had a large contact area but also produced the most significant shear force on the soil. Thus, it may generate greater traction in actual operations.

## 1. Introduction

In the past few decades, the weight of agricultural machinery has increased due to the increase in the number and intensity of global agricultural operations [1]. Improving the traction efficiency of wheeled agricultural machinery is a primary concern of researchers worldwide [2]. Agricultural tractors are primarily used in field operations, so traction performance is one of the main indicators to measure the performance of drive-wheel tires. Tire tread patterns, with tires being the only part in contact with the road surface, are an indispensable factor affecting tire traction performance. A reasonable tread pattern can ensure the excellent traction performance, wear resistance, and self-cleaning performance of the tire. With the development of agriculture, the mechanical properties of tire treads formed in soil have received increasing attention [3].

The traction force generated by the interaction between the wheel and the ground determines the mobility performance of agricultural implements through the soil. Due to subsidence, agricultural tires running on soft soil must withstand greater resistance. Therefore, reducing soil sinkage is crucial for achieving optimal traction performance.

Many scholars have proposed various tire–ground interaction models for soft ground, which can be categorized into three types: (1) empirical models, (2) semi-empirical models, and (3) numerical models.

Empirical models are simple, computationally fast, and commonly used to assess vehicle mobility on soft ground. These models are based on experimental data and include the WES VCI model [4], the WES mobility number model [5], and the STIREMOD model [6,7]. The empirical approach first identifies key indicators that significantly impact vehicle performance and are easy to measure or identify. Field tests are then conducted with vehicles on soft ground, and the results are used to fit the indicators to vehicle performance. Although empirical models are straightforward and effective tools for evaluating vehicle performance on soft ground, their applicability is limited to conditions that precisely match the test conditions of the tires and ground.

Semi-empirical methods are based on appropriate experiments and involve the mechanical analysis of the wheel–ground interaction to establish simplified approximate formulas. A substantial portion of tire–soft ground contact models fall under this category, with nearly all current semi-empirical models being based on the empirical formulas proposed by Bekker [8] and Wong–Reece [9] (or their modified versions). The Bekker model and the Wong–Reece model are crucial tools for describing tire–soil contact behavior. The Bekker model focuses on basic soil compression and shear characteristics, suitable for general tire–soil interaction analysis. In contrast, the Wong–Reece model extends this foundation, offering a more accurate description of tire performance under complex terrain and motion conditions. However, semi-empirical models based on the Bekker and Wong–Reece models are predominantly steady-state models and cannot dynamically compute tire–soil contact forces, and thus fail to meet the requirements of dynamic tire simulation.

Numerical methods are helpful in understanding and describing soil-cutting processes and soil–tread interaction. Many simulation methods have been established to study tire and soft soil interaction [10]. Soil is composed of tiny particles, and the voids between the particles are filled with water and air [11]. The plastic deformation of soil is mainly caused by the sliding between the particles along the contact surface, which usually undergo significant compression and shear deformation. Field measurements showed variations caused mainly by the soil condition [12]. With the continuous improvement of the computing power of numerical computers, the importance of numerical simulation methods in this field has become increasingly prominent. The Finite Element Method (FEM) and Discrete Element Method (DEM) are the primary numerical simulation tools for studying tire–soil interactions. FEM is a numerical analysis tool based on continuum mechanics theory [13]. It partitions complex structures or geometries into a finite number of elements and utilizes nodes at the boundaries of these elements to transmit mechanical information. The process of FEM involves defining material properties, applying boundary conditions, and analyzing the system’s response by solving a series of linear or nonlinear equations. This method is particularly suitable for addressing tire–soil interaction problems with complex geometries and loading conditions because it provides highly accurate stress, strain, and displacement distribution information. Compared with other methods, FEM has the potential to obtain better accuracy results [14]. This method can reduce test costs and provide accurate, detailed results through appropriate calibration [15]. Unlike continuum numerical methods, such as FEM, DEM is a meshless numerical discontinued method that was originally developed for the field of rock mechanics [16,17,18,19,20,21]. With the advancement of research and technology, DEM has gradually been widely applied to simulate the interactions between soil and tools. This method effectively simulates the behavior of granular materials by calculating the contact and interaction force between particles, particularly in fields such as soil engineering, mining, and agriculture. A primary challenge in using DEM is the calibration of microparameters [22,23]. Microparameters are critical data that describe the internal characteristics of materials, including particle shape and size, friction coefficients, and cohesion. These parameters significantly influence the accuracy of simulation results. Improper calibration may lead to substantial discrepancies between simulation outcomes and real-world conditions. Therefore, ensuring that these microparameters accurately reflect the properties of actual materials is essential for effective simulations. Despite the increasing application of DEM across various fields, there is currently a lack of standardized calibration methods or measurement procedures. Different researchers often employ their own methods to determine the same parameter values, resulting in issues related to the comparability and consistency of results. Due to the absence of unified standards, researchers may introduce significant subjectivity into the selection and calibration of parameters, which, in turn, affects the reliability of the simulation results [24].

In the FE method, the main drawback is its inability to handle large deformations. Therefore, CEL (Coupled Eulerian–Lagrangian), ALE (Arbitrary Lagrangian Eulerian), and SPH (Smoothed Particle Hydrodynamics) [25,26] methods can solve the large deformation and complex nonlinear problems of soil failure [27]. Among them, the ALE method requires mesh movement and adjustment, resulting in high computational complexity. The computational accuracy depends on the mesh quality and mesh adjustment method. SPH is prone to issues with particle spacing non-uniformity, which can lead to accuracy problems and necessitates meticulous adjustment and correction. When dealing with solid boundaries and contact problems, the algorithm becomes more complex [28]. The CEL approach is more appropriate [29]. Initially developed for fluid–structure interaction, the CEL method has been formulated to combine the advantages of the Lagrangian and Eulerian formulations. It allows the user to selectively mesh the components’ analysis according to the modeled physics, with the Eulerian technique used in regions undergoing large deformations and the remaining using the conventional Lagrangian technique. Sun et al. used the CEL method to study the shear interface of the track plate under seafloor sediment [12,30]. The CEL method has found many applications in simulating geotechnical problems, such as in laboratory element testing [31], penetration tests in clays [32], ground improvement applications [33], and water–soil interaction problems [34], among others.

The constitutive relationship of materials is essential in finite element analysis, and selecting an appropriate constitutive model is a prerequisite for accurate and reliable numerical prediction [35]. In ABAQUS (CAE 6.13; Dassault Systèmes, Providence, RI, USA, 2013.), various commonly used soil constitutive models are available, including the Mohr–Coulomb, Drucker–Prager, and Cam–Clay models. The Mohr–Coulomb and Cam–Clay models only apply to ABAQUS/standard, while several other classical models need secondary development. Selecting the soil constitutive model according to the specific task is crucial.

In this study, the pattern on the agricultural tire was simplified into a single-pitch pattern block using the scale reduction method [36,37]. A pressing experiment of the pattern blocks under the soil and a traction experiment under pure sliding were carried out. ABAQUS software was used for modeling and simulation analysis. Considering the large deformation and failure of the pattern blocks in the soil during the downward slip process, the CEL method was used for soil modeling.

## 2. Materials and Methods

### 2.1. Materials

**Figure 1:**
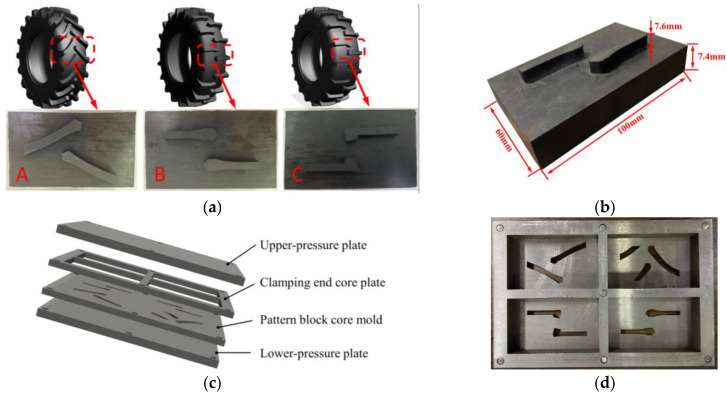
(**a**) Pattern block equivalent model. herringbone (A) pattern blocks, parallel–symmetrical (B) pattern blocks, and parallel–asymmetrical (C) pattern blocks. (**b**) Basic size parameters of pattern block. (**c**) Three-dimensional software modeling pattern block mold. (**d**) Actual pattern block mold.

When studying the interaction mechanism between the tire tread and the road surface, it is unreasonable to produce a complete agricultural tire for experimental testing and evaluate the traction performance of the tire tread in soil. The cost is enormous because this requires a lot of human and material resources. Due to the distribution of the pattern protrusions, a single group of pitch patterns can be selected for analysis. In this study, the pattern on the agricultural tire was scaled to a flat pattern block at a 5:1 ratio for experimental and simulation purposes, and the traction performance of the tire was predicted by local performance [38]. As shown in Figure 1a, three different patterns of agricultural radial tires were selected. The basic size parameters of pattern blocks are shown in Figure 1b.

Due to its high elasticity and high breaking strength, tire tread rubber cannot be formed using tool engraving. A specialized vulcanization mold must be designed to prepare the tread rubber pattern blocks required for the test. In this study, the designed vulcanization mold for the pattern blocks consisted of four parts, as shown in Figure 1c, including the upper-pressure plate, the lower-pressure plate, the pattern block core mold, and the clamping end core mold. The clamping end of the pattern blocks was designed to facilitate the clamping of the pattern blocks in the traction test. The various shapes in the pattern block core mold cavity included herringbone (A) pattern blocks, parallel–symmetrical (B) pattern blocks, and parallel–asymmetrical (C) pattern blocks. They correspond to the left, middle, and right sides of Figure 1a, respectively. A rubber overflow groove was incorporated on the contact surface between the clamping end core mold and the upper-pressure plate to ensure that the excess viscous rubber compound under high temperature and high pressure was discharged, preventing bubbles in the vulcanized pattern blocks. A pinhole was incorporated between the two, and positioning was achieved through a pin connection to prevent the dislocation between the pattern block core mold and the clamping end core mold. The final vulcanization mold is shown in Figure 1d.

**Figure 2:**
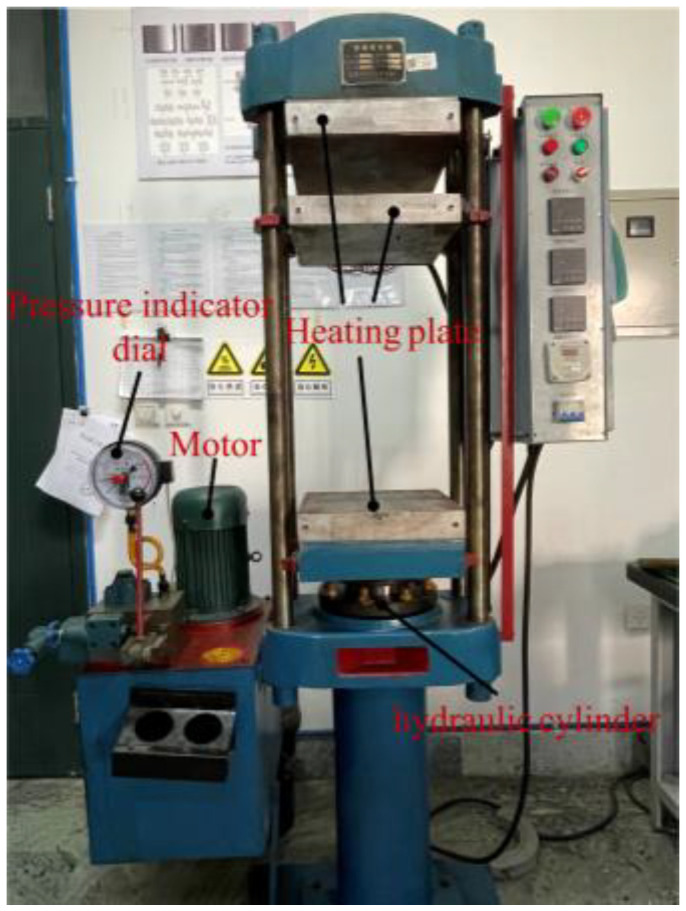
Flatbed vulcanizing machine.

The vulcanization molding process of the pattern blocks primarily consisted of several steps, including the preparation of the rubber compound, the determination of the vulcanization time and temperature, and vulcanization using a flatbed vulcanizing machine (Figure 2).

The preparation of the rubber compound involved placing raw rubber, reinforcing agent, vulcanizing agent, softener, active agent, and other additives into the mixer according to a designated order and then pressing them into tablets by the machine. After the preparation of the rubber compound, it was essential to determine the vulcanizing time and temperature. The vulcanization tester test could obtain the optimum vulcanization time and appropriate vulcanization temperature of the rubber compound. In this experiment, a plate vulcanizing machine was utilized, with the vulcanization pressure set to 15 MPa, the vulcanization temperature at 150 °C, and the working time at 30 min. The final sample of the tread pattern blocks is shown in Figure 1a.

### 2.2. Contact Behavior Testing Methods

**Figure 3:**
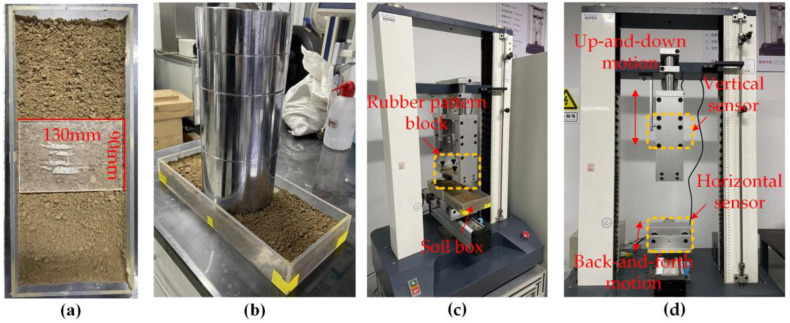
(**a**) Soil bin with cover plate. (**b**) The 30 kg weight compacted soil. (**c**) Compacted soil and pattern blocks placed on the test device. (**d**) Experimental equipment.

A compression movement loading frame (Figure 3d) was utilized to apply stresses to the soil using rubber pattern blocks for the measurement of experimental vertical stress and horizontal traction force. The experimental setup was equipped with two sensors of identical type. One sensor was positioned in the vertical direction to measure vertical load pressure, while the other was positioned in the horizontal direction to measure the traction exerted on the soil trough. A force sensor (model BAB-20MT, Transcell, Buffalo Grove, IL, USA) with a load capacity of 20 kg and a rated output of 2 mV/V was employed to measure the force applied on the sensing surface. The entire testing apparatus could control the specific displacement in the vertical and horizontal directions through the computer. Moreover, the force acting on the tread blocks could be measured by sensors.

The entire experiment could be divided into the following steps: The experimental soil bin had inside dimensions of 450 mm long, 150 mm wide, and 80 mm deep, and the soil surface was smoothed using a flat plate. A panel of 130 mm long, 90 mm wide, and 4 mm deep was placed in the middle of the soil bin (Figure 3a) and a weight of 30 kg (Figure 3b) was applied to it. It was left to stand for 1 min under the load of the weight to ensure the consistency of local soil compaction. Then, the soil groove was fixed on the horizontal position of the test platform (Figure 3d), while the pattern blocks were fixed on the vertical position, as shown in Figure 3c. According to the software operation interface, the force and displacement curve could be measured after setting the depth to 4 mm and the speed to 50 mm/min. After compacting the soil, a horizontal displacement of 10 mm was conducted. Each of the three pattern blocks was measured 6 times, and then data processing and drawing were performed using OriginPro, version 2023 (OriginLab Corporation, Northampton, MA, USA).

## 3. Finite Element Contact Modeling

### 3.1. Constitutive Model

In this research, the Finite Element Method (FEM) model treated soil as a homogeneous material. This meant that the soil was considered to have uniform properties throughout, with consistent parameters applied during treatments. This research employed the Drucker–Prager model for soil simulation. This model is an enhancement of the von Mises model, designed to account for the effects of hydrostatic stress on material failure [39]. By incorporating this factor, the Drucker–Prager model provided a more accurate representation of how soil behaved under various stress conditions, especially in terms of its failure mechanisms. The Drucker–Prager model is particularly suitable for materials that exhibit frictional characteristics, such as soil and rock. It is also applicable in scenarios where the material’s yield point leads to an increase in stiffness. This makes the model versatile and valuable for a wide range of geotechnical engineering applications. The Drucker–Prager model includes multiple failure modes: linear, hyperbolic, and exponential. Among these, the linear failure mode was found to be the most appropriate for modeling soil behavior. This mode provides a balance between complexity and accuracy, making it ideal for practical applications in soil mechanics. Calibration of the Drucker–Prager model can be achieved using results from triaxial stress tests. Alternatively, the model can be calibrated with cohesion and internal friction angle data derived from the Mohr–Coulomb theory [40].

The linear Drucker–Prager criterion was written as Equation (1):

(1) F=t−ptanβ−d=0

In this equation, F is the yield function and t is the deviatoric stress, representing the stress difference in different directions of the material, as given by Equation (2). p is the normal stress, representing the average stress in all directions of the material, as given by Equation (3). β is usually referred to as the material’s friction angle, which is the slope of the linear yield curve in the p−t stress plane and is commonly referred to as the material’s friction angle, reflecting the internal friction characteristics of the material. d is the material’s cohesion. In Equation (2), K represents the ratio of triaxial tensile yield stress to triaxial compressive yield stress. This parameter determines the dependence of the yield surface on the intermediate principal stress value. To ensure the convexity of the yield surface, it is required that 0.778 ≤ K ≤ 1 [40].

(2) $$t=\frac{1}{2}q\left[1+\frac{1}{K}-\left(1-\frac{1}{K}\right){\left(\frac{r}{q}\right)}^{3}\right]$$

(3) $$p=\frac{1}{3}\left(\sigma_{1}+\sigma_{2}+\sigma_{3}\right)$$

(4) $$q=\sigma_{1}-\sigma_{3}$$

To accurately simulate and study the mechanical behavior of soil, it is essential to determine a series of key parameters, including the elastic modulus (E), Poisson’s ratio (v), the Drucker–Prager internal friction angle (β), the flow stress ratio (K), and the dilation angle (ψ). The determination of these parameters typically relies on a series of geotechnical tests conducted in the laboratory, particularly direct shear tests and triaxial tests. Among these tests, the triaxial compression test conducted under consolidated drained conditions can provide more precise and comprehensive data. The direct shear test is mainly used to determine the shear strength parameters of soil, such as cohesion (c) and internal friction angle (β). In contrast, the triaxial test can provide more information about the deformation and strength characteristics of soil under different stress states. In the triaxial test, a complete stress–strain curve could be plotted. When analyzing the stress–strain curve, the elastic modulus (E) could be calculated from the initial tangent slope of the curve. Poisson’s ratio (v) was then calculated using the relationship between the elastic modulus and the shear modulus (G). The shear modulus (G) was calculated as a tangent on the elastic part in the stress–strain curve obtained by the direct shear test [41]. The Drucker–Prager internal friction angle (β) was determined through triaxial testing and described the frictional characteristics of soil under shear conditions. The dilation angle (ψ) described the volumetric change of soil during shear deformation. Estimating the flow stress ratio (K) required combining the results of triaxial tensile and compression tests. By comparing the yield stresses under tensile and compressive conditions, the flow stress ratio of the soil could be determined more accurately. Also, it could be calculated using Equation (5) [42].

(5) $$K=\frac{3-\sin\varphi }{3+\sin\varphi }$$

where φ is the Mohr–Coulomb’s internal angle of friction. The Drucker–Prager internal angle of friction (β) was calculated using Mohr–Coulomb’s internal angle of friction (φ), according to Equation (6) [43].

(6) $$\tan\beta =\frac{6\sin\varphi }{3-\sin\varphi }$$

The linear Drucker–Prager model was selected after evaluating the applicability and convenience of several intrinsic constitutive models. The soil parameters are shown in Table 1.

During the experiment, it was observed that the deformation of the pattern blocks was small. So, the linear elastic model was used in the pattern block simulation modeling. The elastic modulus of the rubber used in the pattern blocks was measured to be E=10 MPa by the tensile test of the material mechanics. Poisson’s ratio was v=0.48. The soil we used was loam for pastoral cultivation, a fine powder obtained after screening, and its physical parameters are shown in Table 2.

### 3.2. Meshing

**Figure 4:**
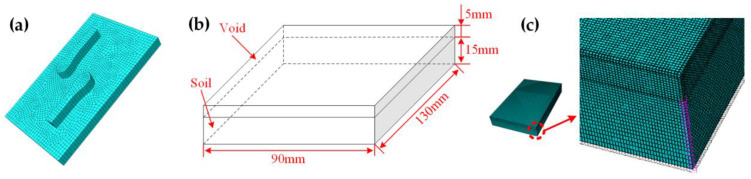
(**a**) B pattern block finite element mesh division. (**b**) CEL structure division of soil. (**c**) Soil finite element mesh division.

The interaction between the pattern block and soil was simulated according to the experimental setup. The rubber pattern block model was established, and its size was the same as the actual parameters. The eight-node linear reduced integration (C3D8R) elements were used to mesh the pattern blocks with the potential for controlling the hourglass. The partitions were meshed with a uniform mesh size of 2 mm. The pattern block model is shown in Figure 4a.

In the case of small deformation, general Lagrange finite element analysis is more accurate. However, for the problem of large deformation, the method is not as accurate as the CEL method [44]. The soil will produce large deformation when agricultural tires are engaged in agricultural production activities. The general contact between the Euler body and the Lagrangian body is used in the CEL algorithm. It is not necessary to specify the primary surface and the slave surface artificially. Thus, the CEL method is suitable for simulating large deformation and nonlinear problems. Therefore, this research method used the CEL method for soil finite element modeling.

The CEL method, which combines the advantages of both the Lagrangian and the Eulerian methods, was implemented in ABAQUS. In numerical analyses using this CEL method, the Eulerian material was tracked as it flowed through the mesh by calculating its Eulerian volume fraction (EVF). Each Eulerian element was designated a percentage, representing the portion filled with material. If an Eulerian element was filled with material, its EVF was 1; if no material existed, its EVF was 0. Contact between Eulerian and Lagrangian materials was enforced using general contact based on a penalty contact method. The Lagrangian elements could move through the Eulerian mesh without resistance until they encountered an Eulerian element filled with material (EVF ≠ 0).

The CEL finite element model for soil simulation was established, as shown in Figure 4b. The Eulerian domain was divided into two regions, the lower part of which was the Eulerian soil area. The area was filled with soil material at the initial simulation time, and its size was 130 mm long, 90 mm wide, and 15 mm deep. The upper part was the Eulerian void area, which was 5 mm higher than the soil model to accommodate the movement and deformation of any Eulerian soil. Initially, the region had no material, mass, or stiffness. The Eulerian domains were divided by a structured grid, and the element type was EC3D8R. The soil profiles were partitioned (Figure 4b). The upper parts were meshed with a 0.60 mm uniform mesh (Figure 4c). Because the intensity of the pattern block contact with the soil surface gradually decreased in the vertical direction of the soil, the mesh size in this direction for the lower parts of the soil increased from 0.6 mm to 1 mm. The whole soil model was divided into 1,562,400 elements. This approach significantly reduced storage requirements and run time due to the coarse mesh used outside the contact area.

### 3.3. Load and Boundaries

The general contact surface (explicit) was used to define the regions of contact between the tire and the soil. The mechanical constraint formulation based on the penalty contact method was used to define the interaction. The contact property was “hard” contact for normal behavior and “penalty” for tangential behavior, with a friction coefficient of 0.3. When the pattern blocks were pressed into the soil, the soil material in the filling area beneath the pattern blocks flowed into the void area above, resulting in soil accumulation.

A reference point was created at the center of the upper surface of the pattern blocks, and the upper plate of the pattern blocks and the reference point (RP) were set as the rigid body, allowing the displacement to be directly applied to the RP in the LOAD module.

**Figure 5:**
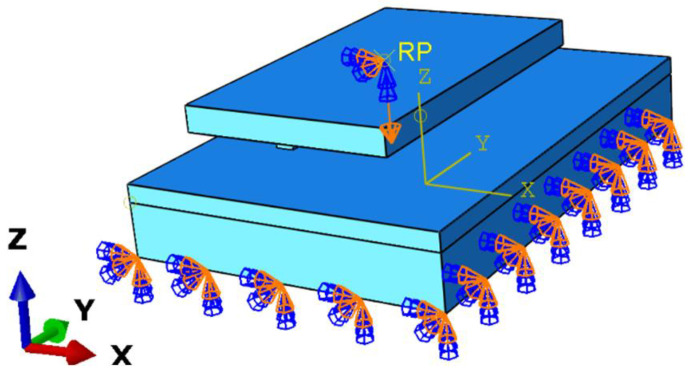
Boundaries of soil and pattern blocks.

The analysis step adopted the dynamic explicit approach. In the first step, the vertical downward displacement of 9 mm (including 5 mm through the Eulerian void area) was applied on the pattern block’s RP (Figure 5) for 0.1 s, during which, only the pattern blocks were allowed to move in the Z (vertical) direction. In the second step of the analysis, the pattern blocks were only allowed to move in the X (horizontal) direction, when a horizontal displacement of 10 mm was applied to the pattern block’s RP for 1 s. Throughout the analysis, the bottom surface of the soil profile was constrained in all directions.

## 4. Results and Discussion

### 4.1. Compaction Process

**Figure 6:**
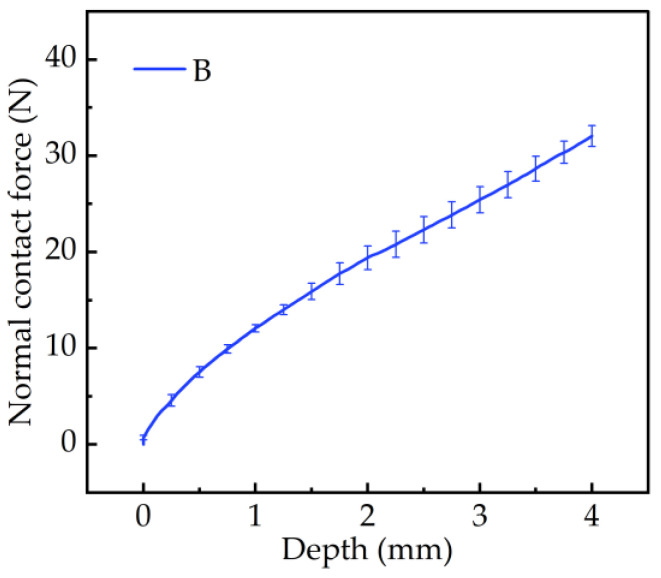
The relationship between the sinking depth and normal contact force for the B pattern block in the test.

**Figure 7:**
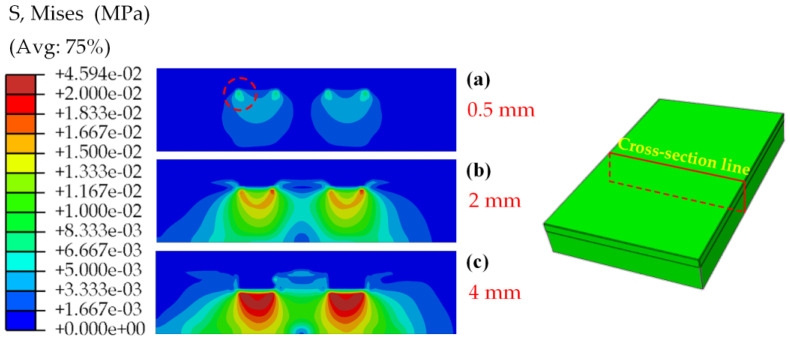
Soil stress in the middle vertical cross-section when the B pattern block was down depths of 0.5 mm (**a**), 2 mm (**b**), and 4 mm (**c**) in the soil.

Figure 6 shows the relationship between the sinking depth of the B tire tread pattern blocks in the soil and the normal contact force. The resulting curve exhibited an almost linear trend. As the depth of downward pressure increased, the normal force acting on the B pattern block also increased. The test results were consistent with the whole-tire soil compaction performed by Hao Li et al. [45]. The simulation diagram (Figure 7) indicates that when the patterned block made contact with the soil, the edge (in the red circles in Figure 7) experienced stress first, and, as the downward pressure was applied gradually, the stress increased from the edge and propagated downward.

### 4.2. Shear Slip Process

When an actual tire moves on soil, the tire is embedded in the soil through a prominent pattern to provide traction for agricultural vehicles. Therefore, when analyzing traction, it is crucial to study the shear force of pattern blocks on the soil.

**Figure 8:**
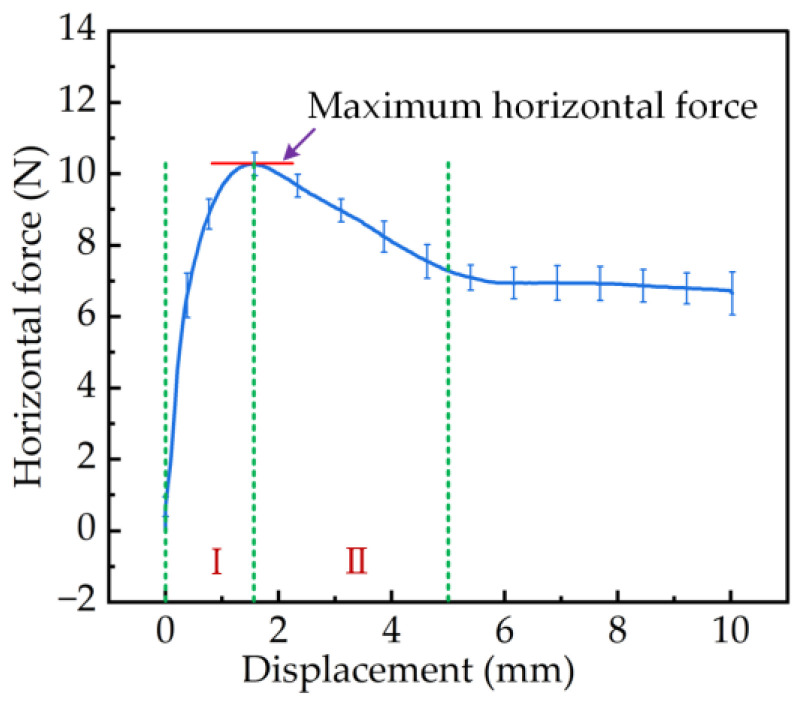
The relationship between the horizontal force on the patterned block and the slip displacement after the B pattern block was pressed 4 mm into the soil in the test.

As shown in Figure 8, after the soil was compressed by 4 mm, the displacement–horizontal force curve was obtained by displacement of 10 mm in the horizontal direction. 

The curve rose sharply, reaching a peak (interval I), then gradually decreased to a specific value (interval II) and eventually leveled off. The maximum horizontal force at the peak of this curve was defined as the soil shear force. The soil was destroyed when the soil shear force reached this critical value.

**Figure 9:**
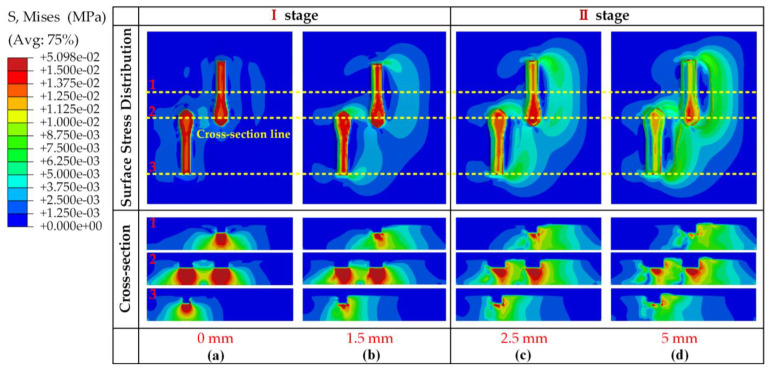
Soil stress distribution on the surface and in the middle vertical cross-section with B pattern block slip displacements of 0 mm (**a**), 1.5 mm (**b**), 2.5 mm (**c**), and 5 mm (**d**) in the soil. Where, the yellow dotted line represents this cross-section, the number represents the cross-section corresponding to the profile.

In interval I, shown in Figure 8, the pattern blocks that had just penetrated the soil began to move horizontally. At this stage, the patterned block compacted the soil, with the maximum stress distributed at the patterned block–soil interface, observed to diffuse downward at the interface as the cross-section (Figure 9a). When reaching the 1.5 mm position, the maximum stress distribution at the pattern block–soil interface and in the cross-section tended to move forward (Figure 9b). The maximum horizontal force at this point may have been due to the pattern blocks’ need to resist their rigid deformation as well as the deformation failure of the soil. Furthermore, when the critical shear force value of the soil was reached, interval II began, the soil began to collapse, and the horizontal force on the pattern blocks decreased. As shown in Figure 9c,d, the surface stress propagated along the direction of the advancement of the pattern block and increased gradually. In the cross-section, it was observed that the stress on the raised side of the pattern block increased with the soil and the stress distribution changed from spreading vertically downward to distributing along the direction of pushing the soil. As the pattern blocks slid a certain distance, the soil damage caused by the reaction force and the stiffness deformation of the pattern blocks reached the equilibrium point and the horizontal force became nearly constant.

### 4.3. Effect of Pattern on Contact Behavior

#### 4.3.1. Contact Behavior in the Compaction Process

**Figure 10:**
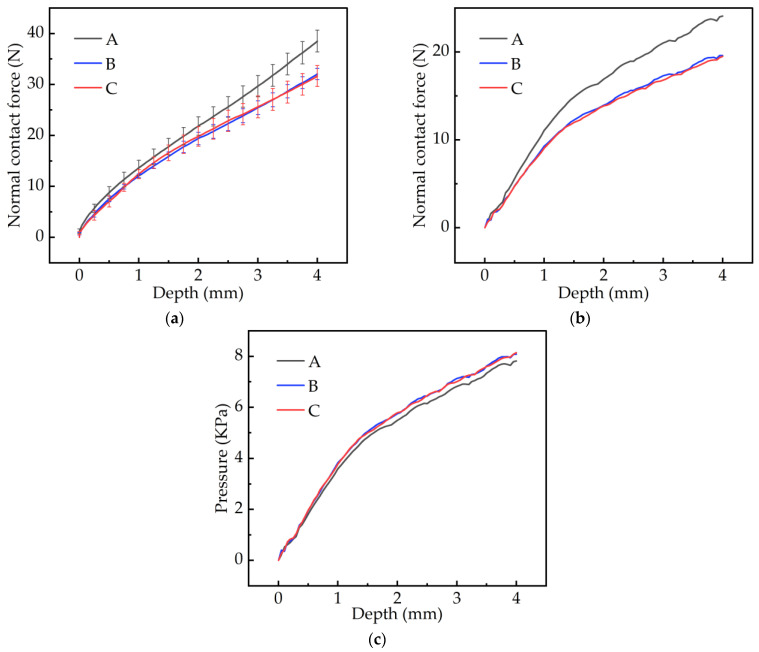
The relationship between sinking depth and normal contact force (**a**) in the test and (**b**) in the simulation. (**c**) Unit contact pressure in the simulation.

Figure 10a,b show the relationship between the sinking depth and normal contact force in the test and simulation. The three pattern blocks produced different force–displacement curves under a loading displacement of 4 mm and a loading speed of 50 mm/min. Although the simulation results showed some discrepancies when compared to the experimental data (Figure 10a), the overall trend remained consistent.

The curve in the experimental test was nearly linear, and the slope of the pattern blocks A, B, and C were 8.48 N/mm, 7.11 N/mm, and 7.10 N/mm, respectively. Among them, the curve of the A pattern was steeper. This indicated that under the same load, the A pattern block caused the least settlement degree to the soil. The B and the C pattern blocks exhibited nearly identical behavior under the same load, which meant that the damage to the soil was also greater. After measuring the actual contact area between the pattern blocks and the soil, it was found that the A pattern block was 3080.6 mm^2^, the B pattern block was 2425.1 mm^2^, and the C pattern block was 2395.8 mm^2^. Dividing the normal contact force in the simulation by the contact area showed that the pressure curves generated by the three pattern blocks were all nearly identical, as shown in Figure 10c. Therefore, increasing the contact area between the tire and the soil could help reduce soil compaction and sinking. As shown in Figure 10a,b, the A pattern block was subjected to the highest stress for the same depth of downward pressure, indicating that it had the best compaction effect on the soil.

**Figure 11:**
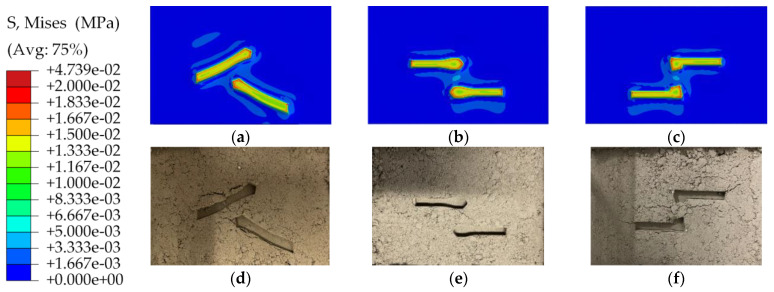
The comparison between the simulation (**a**–**c**) and experimental (**d**–**f**) results of three pattern blocks after a 4 mm deep vertical downpress.

Figure 11a–c show the distribution of von Mises stress and the contour plot of soil deformation. The von Mises stress value of soil compaction deformation is shown in the separate color bar beside the contact footprint. The investigation indicated that the difference in pattern blocks affected soil deformation and soil sinkage. The stress cloud diagram depicts the stress distribution. The red area in the contour indicates regions of high stress. When the tire exerted force on the soil, the maximum stresses were distributed on the raised edges of the tread pattern blocks. The results were consistent with the cross-sectional stress distribution of Figure 7. Rounding the raised edges of the pattern resulted in a more uniform stress distribution and less impact on soil compaction. Figure 11d–f is a diagram of the compaction deformation of the soil in the experiment, which was used to make a comparison with the simulation results.

#### 4.3.2. Contact Behavior in the Shear Slip Process

**Figure 12:**
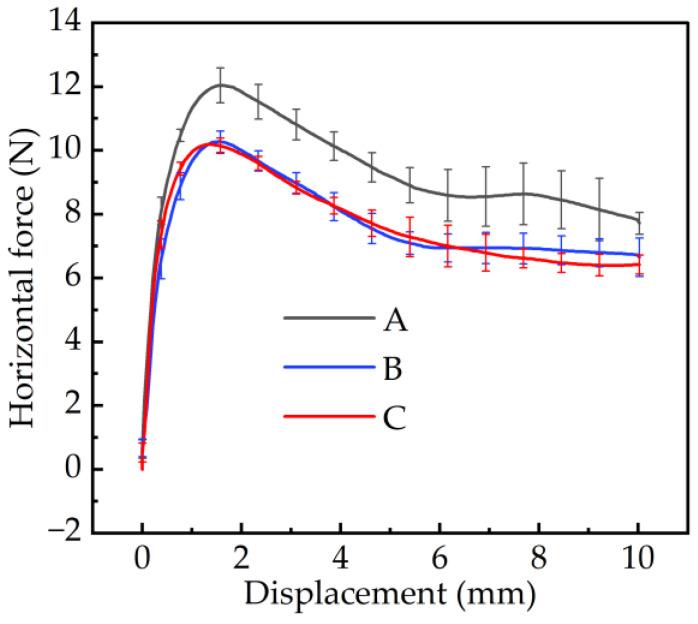
The relationship between the horizontal force and the displacement after three pattern blocks were pressed 4 mm into the soil.

As shown in Figure 12, the displacement–horizontal force curves were measured with 10 mm horizontal displacement of the three pattern blocks. Among the three curves, the soil shear force produced by the A pattern block was the greatest, reaching 12.03 N. The B and C pattern blocks were almost the same, at 10.19 N and 10.27 N, respectively.

When the A pattern block moved forward, the effective contact area with the soil increased, resulting in a larger horizontal force generated at the same slip displacement. Therefore, the A pattern block had better traction performance than the other two under the same soil conditions. Pattern blocks B and C exhibited identical contact areas with the soil. However, despite the different shapes of their central ends, they generated an equivalent horizontal force, indicating that the end shape was not a decisive factor influencing the traction of the pattern blocks.

**Figure 13:**
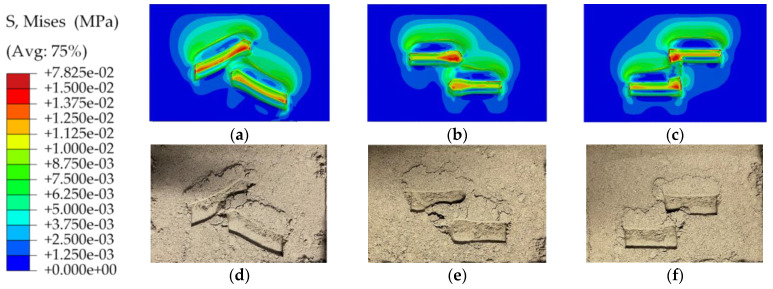
The comparison between the simulation (**a**–**c**) and experimental (**d**–**f**) results of three pattern blocks after 10 mm of horizontal movement.

As shown in Figure 13, the maximum Miss stress occurred at the edge of the bottom of the pattern bump, like the tip of the cutting tool.

**Figure 14:**
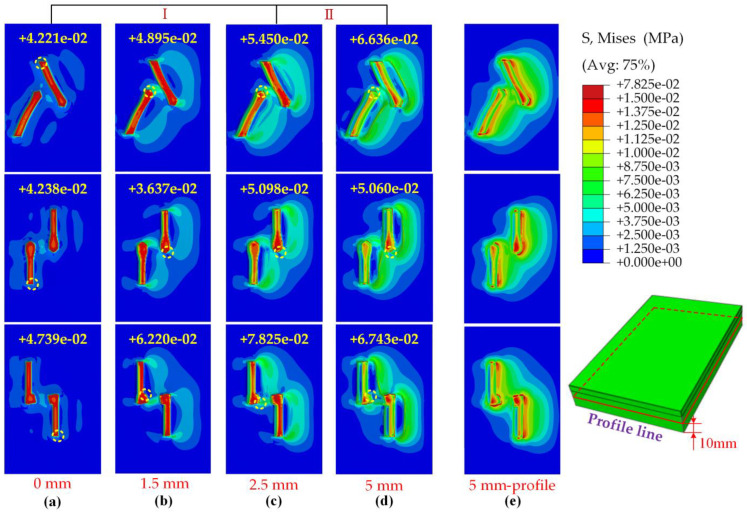
Soil stress contours and max stress value for the three pattern blocks after slip displacement of 0 (**a**), 1.5 (**b**), 2.5 (**c**), and 5 mm (**d**); (**e**) shows the semi-profile of soil after moving 5 mm (profile 10 mm up from the low end). Where the yellow circle represents the position of the maximum stress, and the number represents the maximum stress value.

**Figure 15:**
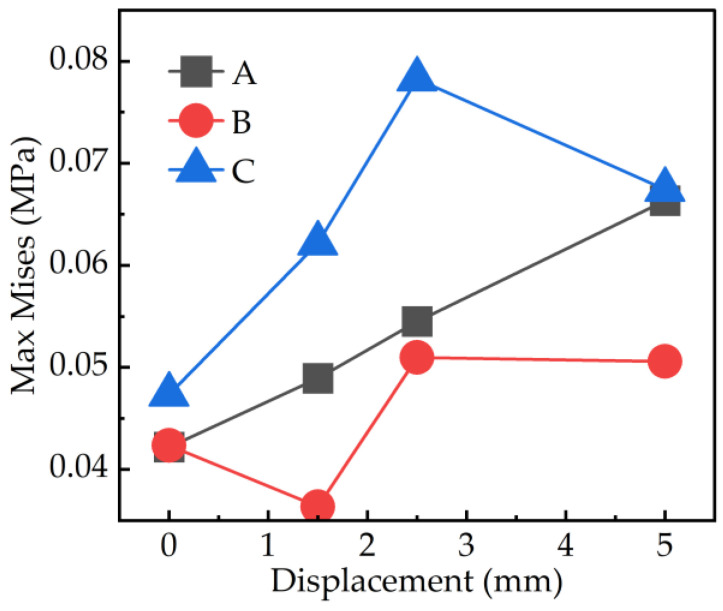
The maximum stress variation with slip distance for three types of tread blocks.

From Figure 14, it is evident that as the slip distance increased, the maximum stress experienced by the soil changed, and the location of this maximum stress also shifted. Figure 15 illustrates the maximum stress experienced by each type of tread block at different positions. As observed, the maximum stress for the A tread blocks increased with the slip distance, whereas the maximum stress for the B and C blocks exhibited irregular variations. These patterns were closely related to the shapes of the three types of tread blocks. However, the locations where the maximum stress occurred were quite similar, typically situated at the part of the tread in contact with the soil on the side facing the direction of movement.

Elastoplastic deformation and plastic failure had a significant influence on the shear process of the pattern blocks. Based on this analysis, the typical shear process occurring at the soil surface can be described as follows.

In the initial shear stage, the soil began to deform, with energy loss primarily attributed to elastic deformation, thereby characterizing this phase as the elastic deformation process. As the shear displacement increased, the soil underwent plastic deformation, gradually resulting in the formation of a shear soil mass. During this phase, the primary energy loss occurred due to plastic deformation. Upon reaching the shear peak, the shear soil began to fracture, with shear stress approaching the residual stress value, resulting in the complete formation of the sliding soil mass. Further analysis of the soil stress distribution and contour extraction is illustrated in Figure 14. Soil stress initially propagated as a stress core and increased in range as the pattern blocks advanced. The highest soil stress was observed at the forefront of the pattern blocks (Figure 14e). Soil uplift was induced by the compression of soil near the pattern blocks, with the kinetic energy of the pattern blocks gradually converting into the potential energy of the soil uplift.

## 5. Conclusions

In this study, by unfolding the tread arc of the tire into a plane and simplifying the large tire into smaller tread blocks, an analysis of tread characteristics was facilitated, significantly reducing the difficulty of the experiments and simulations and lowering costs. The traction performance of the treaded tire was reflected by analyzing the maximum horizontal force generated by different tread blocks during soil slippage. The effects of different agricultural tire tread pattern blocks were investigated through dynamic tests using experimental tests and FEM analysis. In these tests, normal contact pressure and soil shear force were measured. The simulation results demonstrated that the predicted soil deformation and draft force were in reasonable agreement with experimental data. Based on the analysis carried out for the pattern blocks and the specified soil, the following conclusions can be drawn:

1. When a pattern block was pressed into the soil, the stress in the soil increased from the edge and propagated downward. The horizontal force on the patterned blocks was mainly affected by the soil shear force during slipping.
2. With the downward of the pattern blocks, the Normal contact force-depth curve slope of the A, B, and C pattern blocks were 8.48 N/mm, 7.11 N/mm, and 7.10 N/mm, respectively. That means at the same pressure depth, the A pattern block had the greatest contact force and the B and C pattern blocks had very similar contact forces. Thus, the A type performed better in soil compaction.
3. When moving horizontally, the pattern blocks needed to first overcome the force of the destroyed soil. Among the three pattern types, the shear force generated by the A pattern block was the greatest, approximately 17.94% greater than the other two types, which meant that the A pattern block in the actual tire could generate more excellent traction.
4. The proposed CEL method provides new possibilities for simulating soil–tire interaction, which can contribute to a better understanding of the process of interaction between soil and tire patterns and improve the design of tire tread patterns.

## Figures and Tables

**Table 1:** Soil parameters under the Drucker–Prager model.

Parameter Name	Parameter Value
Young’s Modulus (MPa)	0.10
Poisson’s Ratio	0.35
Density (kg/m^3^)	1700
Angle of Friction (°)	26
Flow Stress Ratio	0.861
Dilation Angle (°)	0
Yield Stress (MPa)	0.01

**Table 2:** Physical parameters of soil.

Parameter Name	Parameter Value
Water content (%)	13.04
Granular diameter (mm)	0.005–0.01

## Data Availability

Data can be obtained from the authors upon request.
